# Phenolic Compound, Antioxidant, Antibacterial, and In Silico Studies of Extracts from the Aerial Parts of *Lactuca saligna* L.

**DOI:** 10.3390/molecules29030596

**Published:** 2024-01-25

**Authors:** Aziz Bouymajane, Fouzia Rhazi Filali, Soumia Moujane, Yassine Oulad El Majdoub, Philipp Otzen, Souhail Channaoui, Abdelaziz Ed-Dra, Toufik Bouddine, Khalid Sellam, Ali Ait Boughrous, Natalizia Miceli, Ammar B. Altemimi, Francesco Cacciola

**Affiliations:** 1 Biology, Environment and Health Team, Faculty of Sciences and Technologies, Moulay Ismail University, Meknes 50070, Morocco; 2 Team of Microbiology and Health, Laboratory of Chemistry-Biology Applied to the Environment, Faculty of Sciences, Moulay Ismail University, Meknes 50070, Morocco; 3 Biochemistry of Natural Substances, Faculty of Science and Techniques, Moulay Ismail University, Errachdia 50003, Morocco; 4 Department of Chemical, Biological, Pharmaceutical and Environmental Sciences, University of Messina, 98168 Messina, Italy; 5 Institute of Anorganic and Analytical Chemistry, University of Münster, Corrensstraße 48, 48149 Münster, Germany; 6 Oasis System Research Unit, Regional Center of Agricultural Research of Errachidia, National Institute of Agricultural Research, P.O. Box 415, Rabat 10090, Morocco; 7 Laboratory of Engineering and Applied Technologies, Higher School of Technology, M’ghila Campus, Sultan Moulay Slimane University, Beni Mellal 23000, Morocco; 8 Bioactive Molecules, Health and Biotechnology, Centre of Technology and Transformation, Faculty of Sciences, Moulay Ismail University, Meknes 50070, Morocco; 9 Food Science Department, College of Agriculture, University of Basrah, Basrah 61004, Iraq; 10 Department of Biomedical, Dental, Morphological and Functional Imaging Sciences, University of Messina, 98125 Messina, Italy

**Keywords:** *Lactuca saligna* L., phenolic compounds, willow leaf lettuce, HPLC-PDA/ESI-MS, antioxidant activity, antibacterial activity, ADMET, molecular docking, Morocco

## Abstract

Medicinal plants are considered a major source for discovering novel effective drugs. To our knowledge, no studies have reported the chemical composition and biological activities of Moroccan *Lactuca saligna* extracts. In this context, this study aims to characterize the polyphenolic compounds distributed in hydro-methanolic extracts of *L. saligna* and evaluate their antioxidant and antibacterial activities; in addition, in silico analysis based on molecular docking and ADMET was performed to predict the antibacterial activity of the identified phenolic compounds. Our results showed the identification of 29 among 30 detected phenolic compounds with an abundance of dicaffeoyltartaric acid, luteolin 7-glucoronide, 3,5-di-*O*-caffeoylquinic acid, and 5-caffeoylquinic acid with 472.77, 224.30, 196.79, and 171.74 mg/kg of dried extract, respectively. Additionally, antioxidant activity assessed by DPPH scavenging activity, ferric reducing antioxidant power (FRAP) assay, and ferrous ion-chelating (FIC) assay showed interesting antioxidant activity. Moreover, the results showed remarkable antibacterial activity against *Escherichia coli*, *Salmonella typhimurium*, *Pseudomonas aeruginosa*, *Enterococcus faecalis*, *Staphylococcus aureus*, and *Listeria monocytogenes* with minimum inhibitory concentrations between 1.30 ± 0.31 and 10.41 ± 0.23 mg/mL. Furthermore, in silico analysis identified three compounds, including Apigenin 7-*O*-glucuronide, Quercetin-3-*O*-glucuronide, and 3-p-Coumaroylquinic acid as potent candidates for developing new antibacterial agents with acceptable pharmacokinetic properties. Hence, *L. saligna* can be considered a source of phytochemical compounds with remarkable activities, while further in vitro and in vivo studies are required to explore the main biological activities of this plant.

## 1. Introduction

In recent years, there has been growing interest in exploring natural sources of bioactive compounds with potential health benefits [1,2]. Plants have been recognized as rich reservoirs of various phytochemicals, including phenolic compounds, which have attracted attention for their diverse biological activities [3,4]. Evaluating their antioxidant and antimicrobial properties is crucial to harness their therapeutic potential effectively. Among the wide variety of plant species, *Lactuca saligna*, commonly known as wild lettuce, stands out as a promising candidate for investigation.

*L. saligna*, a member of the Asteraceae family, is widespread throughout the Mediterranean basin and extends into the Caucasus and temperate Europe as far as central Germany and southern Russia, as well as Iraq, Iran, Saudi Arabia, and northern Africa [5,6]. In Morocco, it is widely distributed in different ecological niches and has been traditionally used in folk medicine for its potential therapeutic benefits. However, a comprehensive investigation of the phenolic composition, antioxidant potential, antibacterial activities, and molecular interactions of extracts from the aerial part of *L. saligna* is still lacking.

Phenolic compounds, as secondary metabolites in plants, have been extensively studied for their antioxidant properties and play a pivotal role in combating diseases associated with oxidative stress [7,8,9]. Removal of reactive oxygen species (ROS) involves a variety of mechanisms, including both enzymatic processes (catalase, peroxidases, superoxide dismutase, glutathione reductase, and minerals, which act as enzymatic cofactors, such as copper, iron, and zinc) and non-enzymatic pathways (vitamins B, C, and E as well as phenolic compounds, flavonoids, carotenoids, and α-tocopherols) [10,11,12]. In response to the potential side effects associated with synthetic antioxidants such as butylated hydroxytoluene (BHT), butylated hydroxyanisole (BHA), propyl gallate (PG), ethylenediaminetetraacetic acid (EDTA), and nordihydroguaiaretic acid (NDGA), there is a growing demand for plant-based alternatives [13,14]. The safer nature of plant-based antioxidants is driving their increased use, in line with a broader trend towards natural and sustainable approaches to health and well-being. This paradigm shift highlights the importance of exploring and incorporating the rich array of bioactive compounds found in plants as effective alternatives in the prevention and management of oxidative stress-related diseases.

The global health threat posed by the emergence of multidrug-resistant bacteria is becoming increasingly critical, rendering antibiotics less effective in combating infections [15]. As a response to this growing crisis, numerous plant-derived natural products have surfaced as potent and promising antimicrobials [16,17,18]. In this context, polyphenols derived from medicinal and food herbs have gained prominence as a potential source of effective antioxidant and antimicrobial agents [19]. Scientists and consumers alike are increasingly drawn to polyphenols due to their abundance in our diet, notable antioxidant properties, and crucial role in preventing various diseases associated with oxidative stress, including cancer, cardiovascular diseases, and neurodegenerative conditions [19,20]. The rising interest in polyphenols stems from their recognized capacity to act as antioxidants through mechanisms such as reducing agents, hydrogen donors, singlet oxygen quenchers, and metal chelators [21,22]. This multifaceted role positions phenols as key players in the pursuit of novel strategies for disease prevention and underscores their potential as valuable contributors to global health initiatives.

Recently, in silico studies, including molecular docking and virtual screening, have become indispensable tools in modern drug discovery [23]. These computational approaches can provide insights into the potential interactions between bioactive compounds and target proteins, aiding in the prediction of their pharmacological activities. Integrating in silico studies into the investigation of *L. saligna* extracts can enhance our understanding of the molecular mechanisms underlying their bioactivity.

The present study aims to characterize the phenolic composition of extracts from the aerial parts of *L. saligna*, evaluate their antioxidant potential and antibacterial activities, and employ in silico methods to predict the interactions between the identified phenolic compounds and selected target proteins of bacteria.

## 2. Results and Discussion

### 2.1. Identification of Phenolic Compounds by HPLC-PDA/ESI-MS

**Figure 1:**
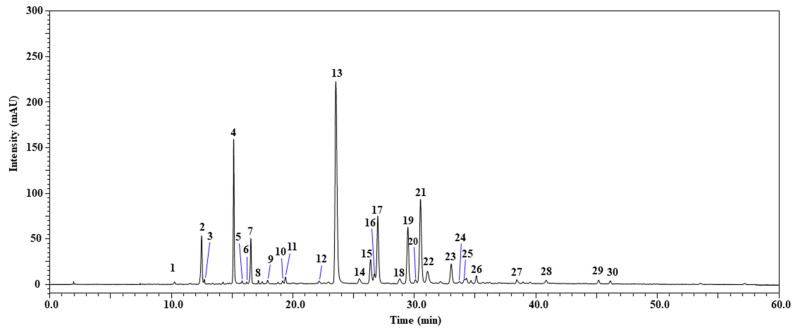
Characterization of phenolic compounds in hydro-methanolic aerial part extract of *L. saligna* acquired at 330 nm using HPLC-PDA/ESI-MS. Peak identification as in Table 1.

The HPLC-PDA/ESI-MS analysis of the phenolic compounds in the studied extracts of *L. saligna* aerial parts led to the detection of 30 compounds (Figure 1); 29 were positively identified according to retention times, λ_max_, mass spectrometry, and literature data, while 1 compound remained unknown (Table 1). The identified compounds were assigned to flavonoids (such as quercetin derivatives, apigenin derivatives, luteolin and its derivatives, genistein, and chrysoeriol) and phenolic acids (caffeic acid and its derivatives, caffeoylmalic acid, caffeoyltartaric acid, dicaffeoyltartaric acid isomers, caffeoylquinic acid isomers, 3-p-Coumaroylquinic acid, di-Hydroxybenzoic acid-hexoside, and Caffeoylferuloylquinic acid). Additionally, the results showed that the most abundant compounds were dicaffeoyltartaric acid (472.77 mg/kg of dried extract), followed by luteolin 7-glucoronide (224.30 mg/kg of dried extract), 3,5-di-*O*-caffeoylquinic acid (196.79 mg/kg of dried extract), and 5-caffeoylquinic acid (171.74 mg/kg of dried extract). To our knowledge, this study is the first to pinpoint phenolic compounds in *L. saligna* extract. Nevertheless, prior studies have detected these compounds within other species of the genus *Lactuca*. Ilgün et al. [24] studied methanolic latex extract from *L. saligna* in Turkey and showed its richness in Lactucin, with 13.94970 ± 0.24 mg_std_/g_latex_. In addition, an Egyptian study revealed the presence of hexacosan-1ol, germanicol, taraxasterol, and moretenol in the aerial parts of *L. saligna* [25]. Hence, it is noted that little is known about the chemical composition of *L. saligna* extract.

### 2.2. Antioxidant Activity

**Figure 2:**
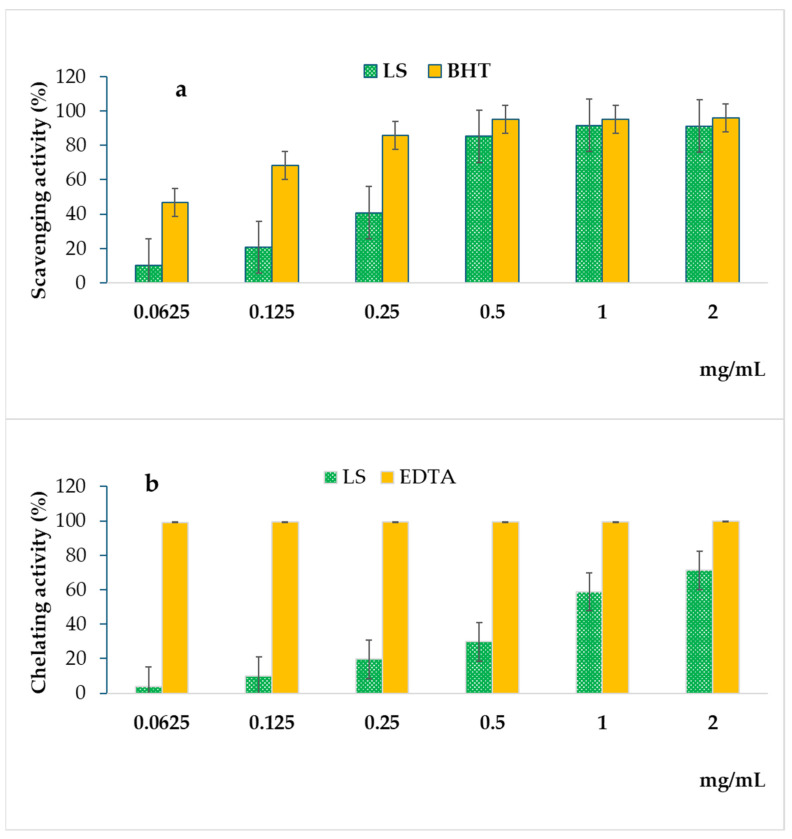
Free radical scavenging activity (**a**) and ferrous ion chelating activity (**b**) of the hydromethanolic extract obtained from the aerial parts of *L. saligna*.

The antioxidant potential of extracts obtained from the aerial parts of *L. saligna* was determined through three distinct in vitro experimental methods, namely, DPPH scavenging activity, ferric reducing antioxidant power (FRAP) assay, and ferrous ion-chelating (FIC) assay. The use of various methods aimed to encompass diverse antioxidant mechanisms present in the plant extract. The obtained results are summarized in Figure 2 and Table 2.

In the DPPH assay, the results revealed a noteworthy radical scavenging activity exhibited by extract derived from *L. saligna*. This activity proved to be highly comparable to that of the standard antioxidant, BHT (butylated hydroxytoluene), particularly within the concentration range of 0.5 to 2 mg/mL (Figure 2a). The observed trend showcased a substantial efficacy of *L. saligna* in neutralizing the stable free radical DPPH, implying a potent capacity for mitigating oxidative stress. Quantitatively, the calculated IC_50_ values further underscored the remarkable performance of *L. saligna* extract in the DPPH assay. In this assay, *L. saligna* showed an interesting antioxidant capacity, with an IC_50_ of 0.2969 ± 0.012 mg/mL compared to the standard BHT with an IC_50_ of 0.0656 ± 0.008 mg/mL (*p* < 0.05) (Table 2). Additionally, the ferric reducing antioxidant power (FRAP) assay showed that *L. saligna* extract exhibits a remarkable antioxidant capacity of 13.952 ± 0.2477 ASE/mL compared to that found in the reference standard (BHT) with a value of 1.131 ± 0.037 ASE/mL (*p* < 0.05). Furthermore, the ferrous ion chelating activity assay showed that *L. saligna* extract has lower antioxidant activity, with an IC_50_ of 1.4219 ± 0.0034 mg/mL compared to the reference standard EDTA (ethylenediaminetetraacetic acid) with an IC_50_ of 0.0067 ± 0.0003 mg/mL (*p* < 0.05) (Figure 2b; Table 2). Consequently, these findings suggest that extract obtained from *L. saligna* holds considerable potential as a natural source of antioxidants, supporting its utilization as a promising candidate for developing nature-based antioxidants for various health and industrial purposes.

To our knowledge, no previous studies have assessed the antioxidant activity of *L. saligna* extracts, especially in Morocco. Notably, our results emphasize the importance of understanding the nuanced antioxidant mechanisms exhibited by plant extracts. While *L. saligna* demonstrated robust radical scavenging activity in the DPPH assay and remarkable performance in the FRAP assay, its performance in the ferrous ion chelating activity assay indicated a less pronounced ability to bind and sequester ferrous ions compared to the reference standard EDTA. Such findings contribute to a comprehensive understanding of the antioxidant profile of *L. saligna* extract, providing valuable information for potential applications and further exploration of its biological activities.

The noteworthy antioxidant activity of *L. saligna* extract can be linked to its chemical composition, notably to its phenolic compounds [32]. In this regard, previous studies have demonstrated the antioxidant activity of dicaffeoyltartaric acid [33], luteolin 7-glucuronide [34], 3,5-di-*O*-caffeoylquinic acid [35], and 5-caffeoylquinic acid [36], which were identified as major phenolic compounds in this study. In addition, flavonoids such as Quercetin derivatives, Apigenin derivatives, Genistein, and Chrysoeriol have shown effective antioxidant activity [37,38,39,40]. Nevertheless, endogenous phenolic compounds may act in synergy to enhance the overall antioxidant activity of extract.

### 2.3. Antibacterial Activity

The antibacterial activity of *L*. *saligna* extract was assessed against six bacterial strains, including Gram-negative and Gram-positive bacteria, by the determination of MIC and MBC values. The obtained results are summarized in Table 3. Our findings based on the determination of MIC values showed that *L*. *saligna* extract is more effective against Gram-positive bacteria (MIC values between 1.30 ± 0.31 and 5.20 ± 0.13 mg/mL) than Gram-negative bacteria (MIC values between 5.20 ± 0.19 and 10.41 ± 0.23 mg/mL). Moreover, the determination of MBC and the ratio between MBC and MIC showed that the studied extract had a bactericidal effect against Gram-positive bacteria and a bacteriostatic effect against Gram-negative bacteria (Table 3).

To our knowledge, no previous studies have assessed the antibacterial activity of *L*. *saligna* extract. Nonetheless, the reason behind it acting differently on Gram-positive and Gram-negative bacteria could stem from distinctions in the cellular structures of these bacteria. Gram-positive bacteria are characterized by a robust layer of peptidoglycan in their cell walls, whereas Gram-negative bacteria possess a thinner peptidoglycan layer in addition to an outer phospholipidic membrane. Similarly, previous studies have demonstrated the high antibacterial activity of plant extract and essential oils against Gram-positive compared to Gram-negative bacteria [8,41].

Notably, the antibacterial activity of plant extracts can be correlated to their chemical composition, with the presence of polyphenolic compounds being a key factor in this correlation [41,42]. In fact, the effective antibacterial activity of *L*. *saligna* extract against both Gram-positive and Gram-negative bacteria may be attributed to its richness in polyphenolic compounds, particularly those belonging to the classes of flavonoids and phenolic acids [43]. Bajko et al. showed that 5-*O*-caffeoylquinic acid has interesting antibacterial activity against both Gram-positive and Gram-negative bacteria, with MICs between 5 and 10 mg/mL [44]. In addition, studies have proven the antibacterial activity of quercetin, apigenin, luteolin, and caffeic acid and their derivative compounds [45,46,47,48]. Interestingly, the mode of action of plant compounds on bacteria is complex and may vary depending on different factors. Hence, it was reported that plant-derived compounds may induce cell wall and membrane rupture, leading to an increase in cellular permeability, the inhibition of proteins involved in septum formation, DNA segregation and cell division, the inhibition of the expression of respiratory chain complex proteins, intracellular ATP depletion, and the disruption of metabolic pathways [41]. Furthermore, plant compounds may exert simultaneous effects at various cellular sites, occasionally demonstrating a synergistic interaction between different compounds that enhances the antibacterial activity of plant extracts.

### 2.4. In Silico Analysis

#### 2.4.1. Virtual Screening

In this study, four molecular compounds, including Apigenin 7-*O*-glucuronide, Quercetin-3-*O*-glucuronide, Chrysoeriol, and 3-p-Coumaroylquinic acid were found to be active against the six bacterial targets. The results of the virtual screening of phenolic compounds extracted from the aerial parts of *L. saligna* are presented in Table 4.

#### 2.4.2. ADMET Analysis

Absorption, distribution, metabolism, excretion, and toxicity (ADMET) play a key role in drug discovery. In our study, the prediction of ADMET properties was performed using the admtsar and pKCSM web servers. The prediction of ADMET parameters is listed in Table 5. This method is very important for selecting the best molecules that we can use as drug candidates. We were interested in Apigenin 7-*O*-glucuronide, Quercetin-3-*O*-glucuronide, Chrysoeriol, and 3-p-Coumaroylquinic acid, the most potent inhibitors in the data set. As a result, we observed that these compounds had high calculated values for intestinal absorption—more than 80%, except for Chrysoeriol, which showed an intestinal absorption of less than 30%, which indicated low absorbance. This indicates that these molecules can be readily absorbed from the gut and circulate in the blood [49]. Distribution analysis showed that the molecules Apigenin 7-*O*-glucuronide, Quercetin-3-*O*-glucuronide, and 3-p-Coumaroylquinic acid are poorly distributed in the brain, with values below 1 [50]. Similarly, metabolizing enzymes are the focus of the main Phase I study in drug discovery. Cytochrome P450 (CYP) includes both substrate and inhibitory enzymes; the most important P450 cytochromes are CYP 2D6 and CYP 3A4 and they are involved in the metabolism of almost half of the drugs currently in use. The results obtained indicate that Apigenin 7-*O*-glucuronide, Quercetin-3-*O*-glucuronide, and 3-p-Coumaroylquinic acid are non-substrates and non-inhibitors of the CYP 2D6 enzyme. However, they are substrates and inhibitors of the CYP 3A4 enzyme.

#### 2.4.3. Molecular Docking

Molecular docking techniques are generally applied to define the binding mechanisms between ligands and receptors. The target ligand is docked to the active site to verify the accuracy of the molecular docking. The molecules with the best virtual screening scores for the six bacteria were docked to the same active sites. Drugs with docking scores between (−98.32 kcal/mol, −102.63 kcal/mol, −88.32 kcal/mol) were selected as promising compounds for *P. aeroginosa*; (−100.1 kcal/mol, −92.16 kcal/mol, −71.21 kcal/mol) were selected for *E. coli*; (−88.25 kcal/mol, −93.48 kcal/mol, −78.18 kcal/mol) were selected for *S. typhimurium*; (−102.36 kcal/mol, −93.16 kcal/mol, −80.63 kcal/mol) were selected for *S. aureus*; (−86.25 kcal/mol, −85.47 kcal/mol, −69.22 kcal/mol) were selected for *E. faecalis*; (−89.03 kcal/mol, −98.62 kcal/mol, −66.61 kcal/mol) were selected for *L. monocytogenes*. The total energy hydrogen bonds (HBonds) and other interactions of the three selected compounds are shown in Table 3.

The results illustrated in Table 3 show that the compounds Quercetin-3-*O*-glucuronide, Apigenin 7-*O*-glucuronide, and 3-p Coumaroylquinic acid are stabilized in the pockets of receptors 1U1Z, 1FJ4, 6IE9, 3JOJ, 6QXS, and 1AOD by various interactions with very low binding affinities of (−7.6, −6.8, −6.5 kcal/mol), (−8.7, −7.9, −7.1 kcal/mol), (−8.4, −8.7, −7.7 kcal/mol), (−8.8, −9.1, −7.6 kcal/mol), (−7.8, −7.7, −7.7 kcal/mol), and (−7.4, −7.8, −6.8 kcal/mol), respectively. Predicted docking results for these three compounds are shown in Supplementary Figures S1–S6.

The molecular docking proved the antibacterial activity of hydro-methanolic extracts of *L. saligna*. Furthermore, the strong binding affinity exhibited by compounds such as Quercetin-3-*O*-glucuronide, Apigenin 7-*O*-glucuronide, and 3-p Coumaroylquinic acid to the specific proteins of the investigated bacteria positions them as promising candidates for the development of novel antibacterial drugs. Nevertheless, additional in vitro and in vivo studies are required.

## 3. Materials and Methods

### 3.1. Chemicals and Reagents

LC-MS grade methanol, formic acid, acetonitrile, and water were purchased from Merk Life Science (Merk KGaA, Darmstadt, Germany). 2,2 diphenyl-1 picrylhydrazyl (DPPH), ethylenediaminetetraacetic acid (EDTA), butylated hydroxytoluene (BHT), potassium ferrycyanide [K_3_Fe (CN)_6_], ferric chloride (FeCl_3_), and trichloroacetic acid were supplied by Carlo Erba (Milan, Italy).

### 3.2. Plant Materials and Phenolic Compound Extraction

The aerial parts of *L. saligna* were harvested in June 2022 from the middle Atlas of Morocco (Ifrane region). The collected plant was identified by the botanist Professor Rahou Abdelilah from the Faculty of Sciences of Meknes, Moulay Ismail University, and confirmed by Professor Ibn Tattou Mohammed at the Scientific Institute of Rabat (Morocco). The plant sample was protected from light and dried at room temperature for 15 days; then, it was crushed and stored at +4 °C until use. The extraction of phenolic compounds was carried out according to our previously published protocol [8].

### 3.3. Polyphenolic Compound Analysis by HPLC-PDA/ESI-MS

The analysis of polyphenolic compounds was performed using high-performance liquid chromatography coupled with a photodiode array detector and electrospray ionization-mass spectrometry (HPLC-PDA-ESI/MS) (Shimadzu, Kyoto, Japan). Chromatographic separation was carried out on an Ascentis Express C18 column (150 × 4.6 mm, 2.7 µm; Merck Life Science, Merck KGaA, Darmstadt, Germany) using as a mobile phase 0.1% (*v*/*v*) acid formic in water (mobile phase A) and 0.1% (*v*/*v*) acid formic in acetonitrile (mobile phase B). The gradient elution applied was as follows: 0–5 min (5% B), 5–15 min (10% B), 15–30 min (20% B), 30–60 min (50% B), and 60 min (100% B) at a flow rate of 0.8 mL/min. The column temperature was 30 °C and the injection volume was 5 µL. UV detection wavelengths were in the range of λ = 100–400 nm. Negative-ion mass spectra were set as follows: scan range, *m*/*z* 100–800; nebulizing gas (N_2_) flow rate, 1.5 L/min; drying gas (N_2_) flow rate, 15 L/min; interface temperature, 350 °C. LabSolutions software ver. 5.92 (Shimadzu, Kyoto, Japan) was used to control the LC-PDA-ESI-MS system and the data processing. The identification of phenolic compounds was performed by a comparison of retention times and UV-visible and mass spectra of unknown peaks with the literature data.

### 3.4. Antioxidant Activity

The determination of the antioxidant activity of *L. saligna* extract was performed based on three different mechanisms, including radical scavenging of DPPH, reducing power (FRAP), and ferrous ions chelating capacity. BHT and EDTA were used as standards for this study.

#### 3.4.1. DPPH Assay

The DPPH scavenging activity of *L. saligna* extract was assessed according to the method described previously [8]. Briefly, 0.5 mL of each concentration of plant extract (0.0625–2 mg/mL) and the standard BHT were mixed with 3 mL of a 0.1 mM methanol DPPH solution and stored in the dark for 20 min. After that, the absorbance of the mixture was determined at 517 nm using a spectrophotometer (UV-1601, Shimadzu). Then, the percentage (%) of radical scavenging activity was calculated using the following formula:

Radical scavenging activity percentage (%) = ((A_0_ − A_c_)/A_0_) × 100

where A_0_ is the DPPH absorbance without the sample and A_c_ is the absorbance in the presence of the sample or standard. The IC_50_ values were calculated as the concentration of extract causing a 50% inhibition of DPPH radical; a lower IC_50_ value corresponded to a higher antioxidant activity of extracts. The experiments were performed in triplicates.

#### 3.4.2. FRAP Assay

The ability to convert Fe^3+^ to Fe^2+^ was used to assess the ferric reducing capacity of *L. saligna* extract, according to the previously described method [51], with few modifications. Briefly, a mixture containing 2.5 mL of phosphate buffer (0.2 M, pH 6.6) and 2.5 mL of 1% potassium ferrycyanide [K_3_Fe (CN)_6_] was prepared and then 1 mL of each sample concentration (0.0625–2 mg/mL) was added. The obtained mixture was incubated at 50 °C for 20 min and then 2.5 mL of 10% trichloroacetic acid was added before the mixture was centrifuged at 3000 rpm for 10 min. Afterward, 2.5 mL of the supernatant was mixed with 2.5 mL of distilled water and 0.5 mL of 0.1% ferric chloride (FeCl_3_) and incubated in the dark for 10 min before measuring the absorbance at 700 nm. The experiments were carried out in triplicates and the obtained results were presented as mean absorbance values ± standard deviation (SD) and acid equivalent (ASE/mL) ± SD.

#### 3.4.3. Ferrous Ions (Fe^2+^) Chelating Activity

The inhibition of Fe^2+^–ferrozine complex formation was used to measure the ferrous ion Fe^2+^ chelating activity of *L. saligna* extract according to the method described previously [8]. Briefly, 1 mL of each concentration of extract (0.0625–2 mg/mL) was mixed with 0.5 mL of methanol and 0.05 mL of 2 mM FeCl_2_; then, the reaction was started by adding 0.1 mL of 5 mM ferrozine and the mixture was incubated in the dark for 10 min at room temperature before measuring the absorbance at 562 nm. EDTA was used as the standard for this assay. The inhibition of ferrozine–(Fe^2+^) complex formation percentage was determined according to the following formula:

Inhibition of ferrozine–Fe^2+^) complex formation (%) = ((A_0_ − A_c_)/A_0_) × 100

where A_0_ is the absorbance of the control and A_c_ is the absorbance in the presence of the sample or standard. The IC_50_ was determined as mean ± SD.

### 3.5. Antibacterial Activity

#### 3.5.1. Bacterial Strains and Culture

To assess the antibacterial activity of *L. saligna* extract, six bacterial species were used in this study, including Gram-negative bacteria—*Escherichia coli*, *Salmonella typhimurium*, and *Pseudomonas aeruginosa*—and Gram-positive bacteria—*Enterococcus faecalis*, *Staphylococcus aureus*, and *Listeria monocytogenes*. Bacterial strains were prepared by sub-culturing a loopful from the frozen stock culture (−80 °C) on Tryptone Soy Yeast Extract Agar (TSYEA; Biolife, Milan, Italy) followed by incubation at 37 °C for 24 h.

#### 3.5.2. The Determination of MIC and MBC

The minimum inhibitory concentrations (CMIs) and minimum bactericidal concentrations (MBCs) were assessed by microdilution assay [51]. In flat-bottom 96-well microplates, the first column wells were used to prepare a mixture of 50 μL of sterile, distilled water and 50 μL of extract (500 mg/mL). From the first well plate, a series of two-fold dilutions were prepared in sterile, distilled water. Afterward, 50 μL of Tryptone Soy Yeast Extract Broth and 50 μL of bacterium suspensions (10^8^ cfu/mL) were added to each well. The positive control contained Tryptone Soy Yeast Extract Broth and a bacterial suspension while the negative control contained Tryptone Soy Yeast Extract Broth without a bacterial suspension. The microplates were incubated at 37 °C for 24 h and then 40 μL of TTC (2,3,5-triphenyl tetrazolium chloride) was added to each well and re-incubated at 37 °C for 30 min. The lowest concentration of extract that did not show bacterial growth was considered as MIC. However, the determination of MBC was performed by sub-culturing 5 μL of wells that did not show bacterial growth on the Tryptone Soy Yeast Extract Agar followed by incubation at 37 °C for 24 h, and the concentrations that did not show any growth of colonies on the media were considered as MBC. Furthermore, the bacteriostatic or bactericidal effect of the extract was determined by calculating the MBC/MIC ratio; if this ratio was below 4, the effect was bactericidal, and if it was greater than 4, the effect was bacteriostatic [52].

### 3.6. In Silico Analysis

#### 3.6.1. Compounds and Bioinformatics Tools

The phenolic compounds identified in *L. saligna* extract by HPLC-PDA/ESI-MS were used for this analysis. The molecular structure of each compound was retrieved from the PubChem database (https://pubchem.ncbi.nlm.nih.gov/, accessed on 9 November 2023), and the optimized compounds were prepared by converting them to pdb format and submitting them in .pdbqt format using Autodock software (http://vina.scripps.edu/, accessed on 9 November 2023). Moreover, RCSB PDB (https://www.rcsb.org/, accessed on 10 November 2023), PyMOL (https://pymol.org/2/, accessed on 15 November 2023), and Discovery Studio (https://www.3ds.com/products/biovia/discovery-studio, accessed on 16 November 2023), were used in this study.

#### 3.6.2. Protein Preparation and Active Site Prediction

The receptors,—(FabZ) (3*R*)-hydroxyacylacyl carrier protein dehydratase (FabZ) (PDB ID: 1U1Z) for *P. aeruginosa* [53], thymidylate synthase (EfTS) (PDB ID: 6QXS) for *E. faecalis* [54], beta-ketoacyl-[acyl carrier protein] synthase (PDB ID: 1fj4) for *E. coli* [55], phosphatidylinositol-specific phospholipase C (PDB ID: 1AOD) for *L. monocytogenes* [56], RamR PDB (6IE9) for *S. typhimurium* [57], and the UDP-N-acetyl-mannosamine dehydrogenase Cap5O (PDB ID: 3JOL) for *S. aureus* [58]—were retrieved from the protein database (PDB) and processed by removing water molecules and any small molecules loaded with the target receptor using PyMOL software, then submitted to autodock tool software to locate the active site in the processed receptor and convert it to (.pdbqt) format by autodock vina for docking calculations.

#### 3.6.3. Structure-Based Virtual Screening

The software iGEMDOCK (Generic Evolution Method for Docking) version 2.1 was used to perform high-speed virtual screening. In silico screening of 29 identified phenolic compounds in *L. saligna* extract was performed using PDB codes (ID: 1U1Z) for *P. aeruginosa*, (ID: 6QXS) for *E. faecalis*, (ID: 1fj4) for *E. coli*, (ID: 1AOD) for *L. monocytogenes*, (ID: 6IE9) for *S. typhimurium*, and (ID: 3JOL) for *S. aureus*. The screening score, which was calculated from the total energy calculations (Total energy = VdW + HBond + electrostatic), was calculated using iGEMDOCK v2.1.11. The standard parameters used for screening, namely, population size, generations, and number of solutions, were set to 300, 70, and 2, respectively. The energy-based results were analyzed, and 4 potential inhibitors were selected based on their stability for more detailed analyses.

#### 3.6.4. ADMET Analysis

As part of the process of developing a new antimicrobial drug, it is essential to assess the pharmacologically active substance, and this assessment was carried out in silico using ADMET (absorption, distribution, metabolism, excretion, and toxicity) analysis; ADMET parameters were calculated using admetASR 2.0 and pkCSM (https://biosig.lab.uq.edu.au/pkcsm/ accessed on 20 November 2023).

#### 3.6.5. Molecular Docking

The anchoring process involved extracting the co-crystallized reference ligand and water molecules from the crystal structure. Polar hydrogen atoms were added. Throughout the anchoring process, the protein was maintained in a rigid state, while the ligand was allowed to be extremely flexible. The ligands (Apigenin 7-*O*-glucuronide, Quercetin-3-*O*-glucuronide, 3-p-Coumaroylquinic) with the best pharmacokinetic properties and virtual screening results were anchored to ((3*R*)-hydroxyacylacyl) carrier protein dehydratase for *P. aeruginosa* (PDB ID: 1U1Z), thymidylate synthase (EfTS) (PDB ID: 6QXS) for *E. faecalis*, beta-ketoacyl-[acyl carrier protein] synthase (PDB ID: 1fj4) for *E. coli*, UDP-N-acetyl-mannosamine dehydrogenase Cap5O (PDB ID: 3JOL) for *S. aureus*, and phosphatidylinositol-specific phospholipase C (PDB 1AOD) for *L. monocytogenes* (Figures S1–S6). Autodock vina was used to generate binding positions for the bioactive ligands in the active sites of the six targets. Once docking was complete, ligand placement was used to obtain the minimum binding energy. Discovery Studio and PyMOL were used to visualize the results. The type of interactions established by each molecule in the active sites was also compared.

### 3.7. Statistical Analysis

Statistical analysis was performed using SPSS software (SPSS version 22, IBM Corp, Armonk, NY, USA). Data were subjected to an analysis of variance, followed by Duncan’s New Multiple Range Test (DMRT). All experiments were performed in triplicate and the differences were considered significant at *p* < 0.05.

## 4. Conclusions

This study reported, for the first time, the polyphenolic compound composition and antioxidant and antibacterial activities of aerial-part extracts of Moroccan *L. saligna*. Interestingly, *L. saligna* can be considered an interesting source of secondary metabolites, where HPLC-PDA/ESI-MS identified 29 among 30 detected compounds. Additionally, hydro-methanolic extracts of *L. saligna* exhibited high antioxidant activity and remarkable antibacterial activity against both Gram-positive and Gram-negative bacteria. Furthermore, in silico analysis showed that among the 29 identified compounds, Apigenin 7-*O*-glucuronide, Quercetin-3-*O*-glucuronide, and 3-p-Coumaroylquinic acid exhibit high binding affinity scores and establish stable interactions. Consequently, our study showed that *L. saligna* is a potent source of biomolecules and could be used as a sustainable source for developing drugs with interesting antioxidant and antibacterial activities.

## Figures and Tables

**Table 1:** Identification of phenolic compounds in hydromethanolic aerial part extract of *L. saligna* using HPLC-PDA/ESI-MS.

N°	Compounds	t_R_ (min)	UVmax (nm)	[M − H]^−^ *m*/*z*	Phenolic Content *	References
1	di-Hydroxybenzoic acid-hexoside	10.30	314	315	-	[26]
2	Caffeoyltartaric acid	12.51	327	311	76.39	[27]
3	Caffeic acid-hexoside	12.73	289	341	9.96	[26]
4	5-Caffeoylquinic acid	15.15	298, 325	353	171.74	[27]
5	Quercetin hexose-glucuronide	15.83	342	639	2.05	[26]
6	Quercetin-*O*-di-hexoside	16.22	339	625, 301	1.38	[26]
7	Caffeic acid	16.57	294, 323	179	66.05	[28]
8	Apigenin glucoside	17.18	355	431	1.03	[28]
9	3-p-Coumaroylquinic acid	17.93	311	337	11.70	[26]
10	Caffeoylmalic acid	19.18	326	295	9.40	[27]
11	Caffeoylferuloylquinic acid	19.41	325	367	16.27	[29]
12	Quercetin-3-*O*-glucuronide	22.19	279, 341	477, 301	3.33	[26]
13	Dicaffeoyltartaric acid	23.55	299, 328	473, 311, 179	472.77	[27]
14	Dicaffeoyltartaric acid isomer	25.48	299, 328	473, 311, 179	16.79	[27]
15	Quercetin hexose	26.40	254, 350	463, 303+	33.96	[28]
16	di-4-Hydroxyphenylacetyl-hexose	26.71	347	447	-	[28]
17	Luteolin 7-glucoronide	27.00	252, 347	461	224.30	[28]
18	p-Coumaroylcaffeoyltartaric acid	28.78	320	457	17.88	[27,28]
19	Quercetin 6-acetyl-3-*O*-glucoside	29.47	255, 354	505, 301	82.36	[30]
20	Quercetin malonylglucoside	30.09	363	549, 505, 303+	8.56	[27]
21	3,5-di-*O*-Caffeoylquinic acid	30.51	297, 326	515, 353, 179	196.79	[26]
22	Quercetin 3-*O*-rhamnoside	31.07	331	447, 301	17.96	[28]
23	Apigenin 7-*O*-glucuronide	33.03	267, 334	445, 269	8.89	[28]
24	Luteolin 7-glucoronide	33.70	341	461	7.92	[28]
25	3,5-di-*O*-Caffeoylquinic acid isomer	34.11	326	515	13.43	[26]
26	Apigenin 7-*O*-glucoside	35.10	267, 282, 346	431	3.59	[31]
27	Unknown	38.42	331	473, 269	-	-
28	Luteolin	40.85	350	285	13.20	[28]
29	Genistein	45.16	331	269	9.63	[31]
30	Chrysoeriol	46.12	345	299	1.52	[31]

* Phenolic content in dried extract (mg/kg); (-) fragments observed; (+) detected in positive ionization mode.

**Table 2:** Antioxidant activity of hydromethanolic extract obtained from the aerial part of *L. saligna*.

Samples	DPPH Assay IC_50_ (mg/mL)	FRAP Assay ASE/mL	FIC Assay IC_50_ (mg/mL)
*L. saligna* extract	0.297 ± 0.012 ^a^	13.952 ± 0.248 ^a^	1.422 ± 0.003 ^a^
BHT	0.065 ± 0.008 ^b^	1.131 ± 0.037 ^b^	ND
EDTA	ND	ND	0.007 ± 0.000 ^b^

Values are expressed as the mean ± SD (n = 3). Different letters within the same column indicate significant differences between mean values (one-way ANOVA followed by Duncan’s New Multiple Range Test, *p* < 0.05). ND: not determined.

**Table 3:** MIC and MBC (mg/mL) exhibited by hydromethanolic extracts obtained from the aerial part of *L. saligna* against pathogenic bacteria. Results presented as mean ± SD.

Bacteria	Gram Type	MIC	MBC	MBC/MIC	Effect
*Escherichia coli*	−	10.41 ± 0.23	83.33 ± 0.12	8	Bacteriostatic
*Pseudomonas aeruginosa*	−	5.20 ± 0.19	83.33 ± 0.20	16	Bacteriostatic
*Salmonella typhimurium*	−	10.41 ± 0.14	166.66 ± 0.12	16	Bacteriostatic
*Listeria monocytogenes*	+	5.20 ± 0.13	10.83 ± 0.12	2	Bactericidal
*Enterococcus faecalis*	+	5.20 ± 0.22	10.83 ± 0.15	2	Bactericidal
*Staphylococcus aureus*	+	1.30 ± 0.31	5.20 ± 0.16	4	Bactericidal

**Table 4:** Docking results showing the binding affinities of phytocompounds and the hydrogen interactions established with amino acids (Figures S1–S6).

	Total Energy kcal/mol	Binding Affinity kcal/mol	Hydrogen Bonds
*Pseudomonas aeroginosa*			
Apigenin 7-*O*-glucuronide	−98.32	−7.6	Trp(60), Tyr(93), Cys(79)
Quercetin-3-*O*-glucuronide	−102.63	−6.8	Tyr(75), Thy(115), Ser(129), Tyr(47)
3-p-Coumaroylquinic acid	−88.32	−6.5	Tyr(93), Ser(129)
*Escherichia coli*			
Apigenin 7-*O*-glucuronide	−100.1	−8.7	Asn(396)
Quercetin-3-*O*-glucuronide	−92.16	−7.9	Leu(9), Asp(25), Asn(17), Glu(80)
3-p-Coumaroylquinic acid	−71.21	−7.1	Gly(391), Thr(302), Gly(205), Met(204), Val(270)
*Salmonella Typhimurium*			
Apigenin 7-*O*-glucuronide	−88.25	−8.4	Thr(85), Cys(67), Asp(152)
Quercetin-3-*O*-glucuronide	−93.48	−8.7	Ser(137), Thr(85), Asp(124)
3-p-Coumaroylquinic acid	−78.18	−7.7	Asp(30), Thr(35), Ser(103)
*Staphylococcus aureus*			
Apigenin 7-*O*-glucuronide	−102.36	−8.8	Thr(82), Cys(258), Asn(84), Asp(30)
Quercetin-3-*O*-glucuronide	−93.16	−9.1	Asn(84), Thr(119), Val(80), Ala(79), Glu(151)
3-p-Coumaroylquinic acid	−80.63	−7.6	Ser(137), Thr(85), Tyr(59)
*Enterococcus faecalis*			
Apigenin 7-*O*-glucuronide	−86.25	−7.8	Glu(59), Pro(195), Ser(218)
Quercetin-3-*O*-glucuronide	−85.47	−7.7	Ile(80), His(198), Asn(228)
3-p-Coumaroylquinic acid	−69.22	−6.7	Asp(30), Thr(35), Ser(103)
*Listeria monocytogenes*			
Quercetin-3-*O*-glucuronide	−89.03	−7.4	Asp(229), Thr(34)
Apigenin 7-*O*-glucuronide	−98.62	−7.8	Asn(207), Leu(62), Glu(128)
3-p-Coumaroylquinic acid	−66.61	−6.8	Asp(204), Asn(207)

**Table 5:** The results of the ADMET test with pKCSM of potent antibacterial compounds from *L. saligna* extract.

Compounds	Apigenin 7-*O*-glucuronide	Quercetin-3-*O*-glucuronide	Chrysoeriol	3-p-Coumaroylquinic Acid
Absorption and Distribution				
Blood–Brain Barrier	−1.305	−1.322	−0.943	−1.16
Human Gut Absorption	67	70	29	82
Substrat glycoprotéine P	-	-	-	-
Inhibitor of Glycoprotein P	-	-	-	-
Metabolism				
CYP450 2D6 Substrate	No	No	No	No
CYP450 3A4 Substrate	Yes	Yes	Yes	Yes
CYP450 2D6 Inhibitor	No	No	No	No
CYP3A4 Inhibitors	No	No	No	No
Excretion and Toxicity				
Hepatotoxicity	No	No	No	No
Carcinogens	No	No	No	No
AMES Mutagenicity	No	No	No	No

## Data Availability

Data are contained within the article and Supplementary Materials.

## Supplementary Materials

The following supporting information can be downloaded at https://www.mdpi.com/article/10.3390/molecules29030596/s1. Figure S1. 2D view of the conformations of different interactions between the three inhibitors in the 1U1Z active site; Figure S2. 2D view of the conformations of different interactions between the three inhibitors in the 1FJ4 active site; Figure S3. 2D view of the conformations of different interactions between the three inhibitors in the 6IE9 active site; Figure S4. 2D view of the conformations of different interactions between the three inhibitors in the 3JOL active site; Figure S5. 2D view of the conformations of different interactions between the three inhibitors in the 6QXS active site; Figure S6. 2D view of the conformations of different interactions between the three inhibitors in the 1AOD active site.
