# Supplementary material for: Phenolic Compound, Antioxidant, Antibacterial, and In Silico Studies of Extracts from the Aerial Parts of Lactuca saligna L
Source: Molecules. 2024 Jan 25;29(3):596. doi: 10.3390/molecules29030596 (PMC10856452; doi:10.3390/molecules29030596)
Supplement: Supplementary file 1 [file molecules-29-00596-s001.zip › molecules-2807390-supplementary.pdf]

Supplementary Data

Phenolic Compound, Antioxidant, Antibacterial, and In Silico Studies of Extracts from the Aerial Parts of *Lactuca saligna* L.

Aziz Bouymajane [1,2,*], Fouzia Rhazi Filali [2], Soumia Moujane [3], Yassine Oulad El Majdoub [4], Philipp Otzen [5], Souhail Channaoui [6], Abdelaziz Ed-Dra [7], Toufik Bouddine [8], Khalid Sellam [1], Ali Ait Boughrous [1],
Natalizia Miceli [4], Ammar B. Altemimi [9] and Francesco Cacciola [10,*]

[1] Biology, Environment and Health Team, Faculty of Sciences and Technologies, Moulay Ismail University, Meknes 50070, Morocco; k.sellam@fste.umi.ac.ma (K.S.); a.aitboughrous@umi.ac.ma (A.A.B.)
[2] Team of Microbiology and Health, Laboratory of Chemistry-Biology Applied to the Environment, Faculty of Sciences, Moulay Ismail University, Meknes 50070, Morocco; fouzia.filali@yahoo.fr
[3] Biochemistry of Natural Substances, Faculty of Science and Techniques, Moulay Ismail University, Errachdia 50003, Morocco; soumiamjn@gmail.com
[4] Department of Chemical, Biological, Pharmaceutical and Environmental Sciences, University of Messina, 98168 Messina, Italy; yassine.ouladelmajdoub@unime.it (Y.O.E.M.); nmiceli@unime.it (N.M.)
[5] Institute of Anorganic and Analytical Chemistry, University of Münster, Corrensstraße 48, 48149 Münster, Germany; philipp.otzen@uni-muenster.de
[6] Oasis System Research Unit, Regional Center of Agricultural Research of Errachidia, National Institute of Agricultural Research, P.O. Box 415, Rabat 10090, Morocco; souhail.channaoui@inra.ma
[7] Laboratory of Engineering and Applied Technologies, Higher School of Technology, M'ghila Campus, Sultan Moulay Slimane University, Beni Mellal 23000, Morocco; abdelaziz_iaa@yahoo.fr
[8] Bioactive Molecules, Health and Biotechnology, Centre of Technology and Transformation, Faculty of Sciences, Moulay Ismail University, Meknes 50070, Morocco; bouddinet@gmail.com
[9] Food Science Department, College of Agriculture, University of Basrah, Basrah 61004, Iraq; ammar.ramddan@uobasrah.edu.iq
[10] Department of Biomedical, Dental, Morphological and Functional Imaging Sciences, University of Messina, 98125 Messina, Italy
* Correspondence: a.bouymajane@edu.umi.ac.ma (A.B.); cacciolaf@unime.it (F.C.)

Abstract: Medicinal plants are considered a major source for discovering novel effective drugs. To our knowledge, no studies have reported the chemical composition and biological activities of Moroccan *Lactuca saligna* extracts. In this context, the study aims to characterize the polyphenolic compounds distributed in hydro-methanolic extract of *L. saligna* and evaluate its antioxidant and antibacterial activity; in addition, *in silico* analysis based on molecular docking and ADMET has been performed to predict the antibacterial activity of the identified phenolic compounds. Our results showed the identification of 29 among 30 detected phenolic compounds with the abundance of dicaffeoyltartaric acid, luteolin 7-glucoronide, 3,5-di-O-caffeoylquinic acid, and 5-caffeoylquinic acid with 472.77, 224.30, 196.79, and 171.74 mg/Kg of dried extract, respectively. Additionally, antioxidant activity assessed by DPPH scavenging activity, ferric reducing antioxidant power (FRAP) assay, and ferrous ion-chelating (FIC) assay showed interesting antioxidant activity. Moreover, the results showed remarkable antibacterial activity against *Escherichia coli*, *Salmonella* Typhimurium, *Pseudomonas aeruginosa*, *Enterococcus faecalis*, *Staphylococcus aureus*, and *Listeria monocytogenes* with minimum inhibitory concentrations between 1.30±0.31 and 10.41±0.23 mg/mL. Furthermore, *in silico* analysis identified three compounds, including Apigenin 7-O-glucuronide, Quercetin-3-O-glucuronide, and 3-p-Coumaroylquinic acid as potent candidates for developing new antibacterial agents with acceptable pharmacokinetic properties. Hence, *L. saligna* can be considered a source for phytochemical compounds with remarkable activities, while further *in vitro* and *in vivo* studies are required to explore the main biological activities of this plant.

Keywords: *Lactuca saligna* L.; Phenolic compounds; Willow leaf lettuce; HPLC-PDA/ESI-MS; Antioxidant activity; Antibacterial activity; ADMET; Molecular docking; Morocco

---

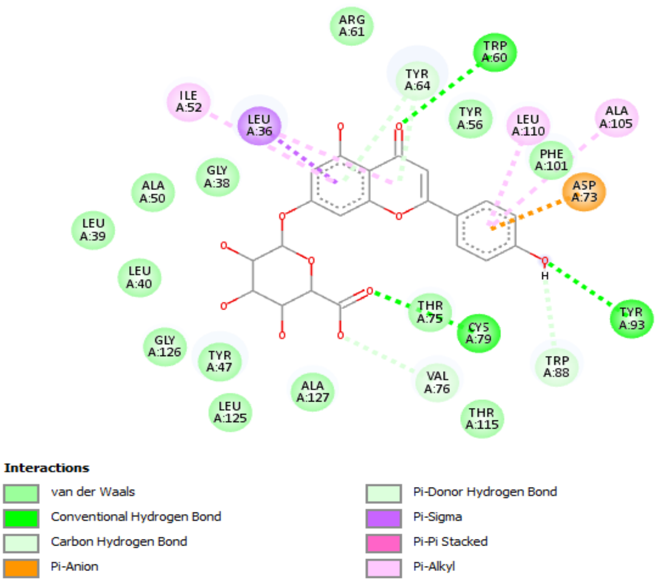

Apigenin 7-O-glucuronide

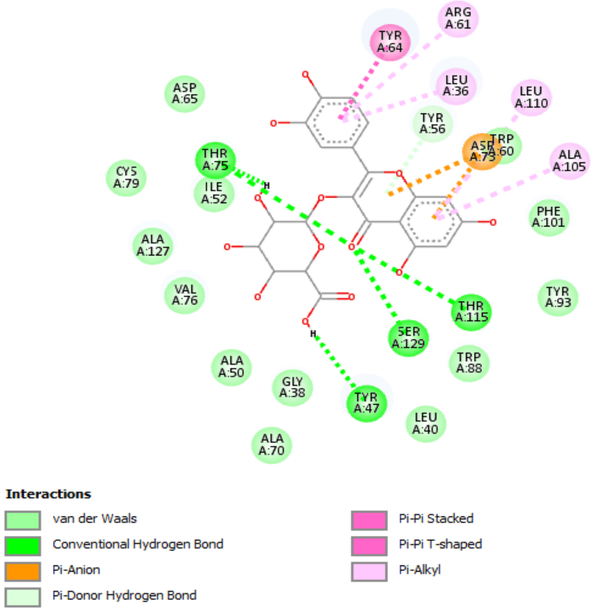

Quercetin-3-O-glucuronide

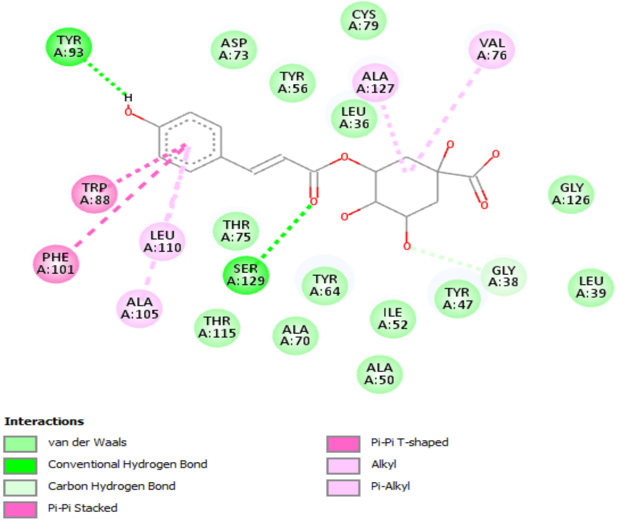

3-p-Coumaroylquinic acid

Figure S1. 2D view of the conformations of different interactions between the three inhibitors in the 1U1Z active site.

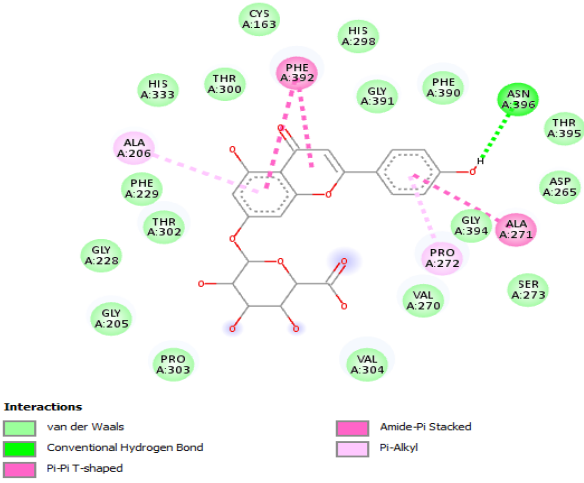

Apigenin 7-O-glucuronide

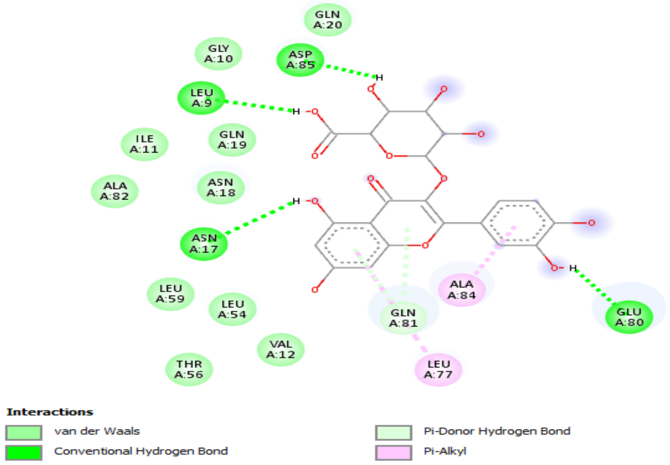

Quercetin-3-O-glucuronide

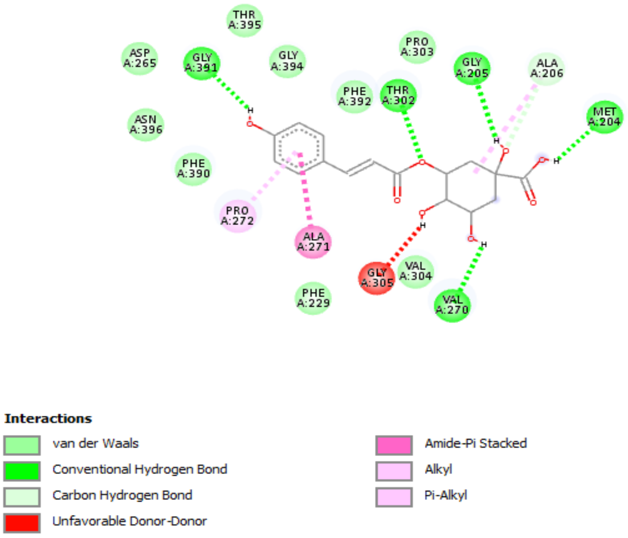

3-p-Coumaroylquinic acid

Figure S2. 2D view of the conformations of different interactions between the three inhibitors in the 1FJ4 active site.

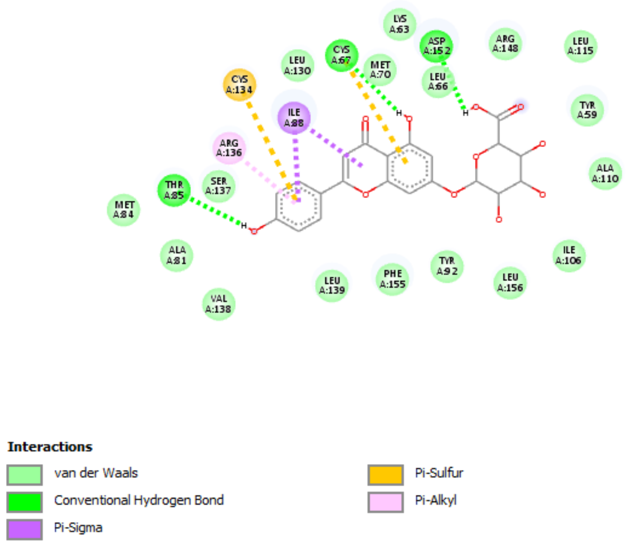

Apigenin 7-O-glucuronide

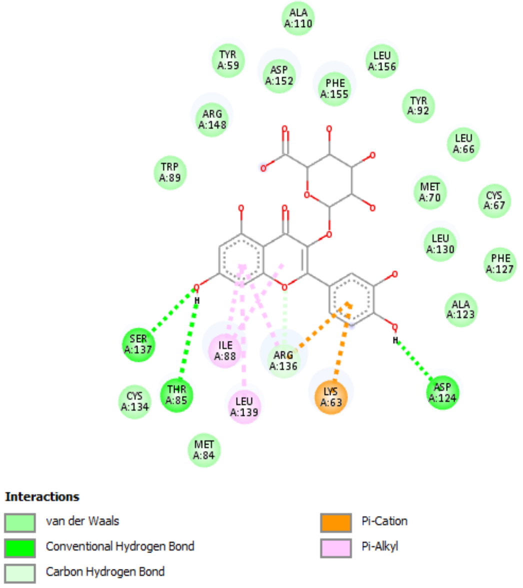

Quercetin-3-O-glucuronide

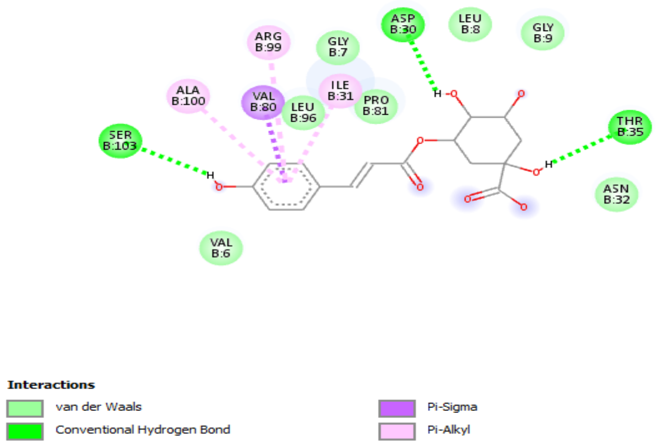

3-p-Coumaroylquinic acid

Figure S3. 2D view of the conformations of different interactions between the three inhibitors in the 6IE9 active site.

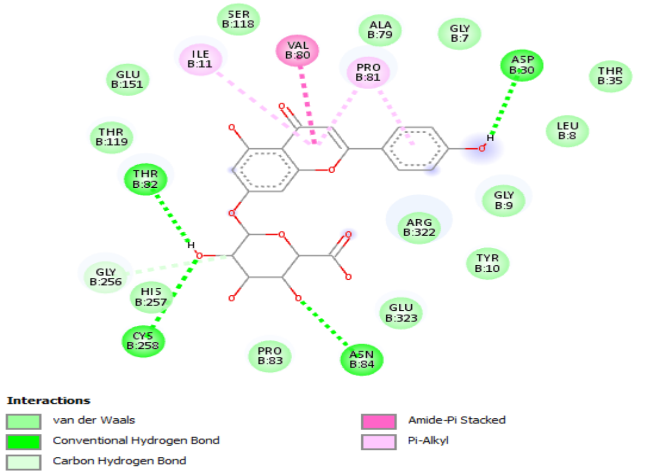

Apigenin 7-O-glucuronide

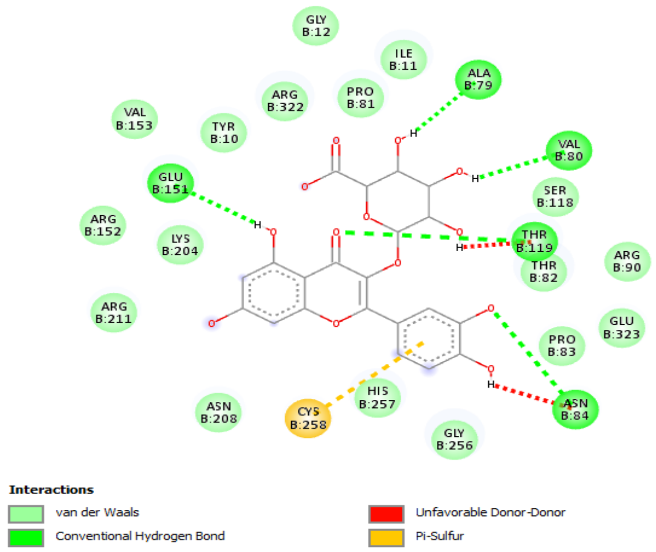

Quercetin-3-O-glucuronide

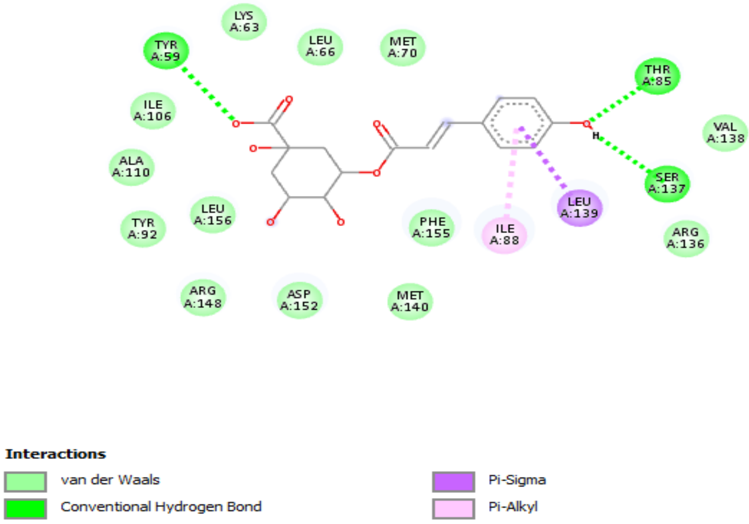

3-p-Coumaroylquinic acid

Figure S4. 2D view of the conformations of different interactions between the three inhibitors in the 3JOL active site.

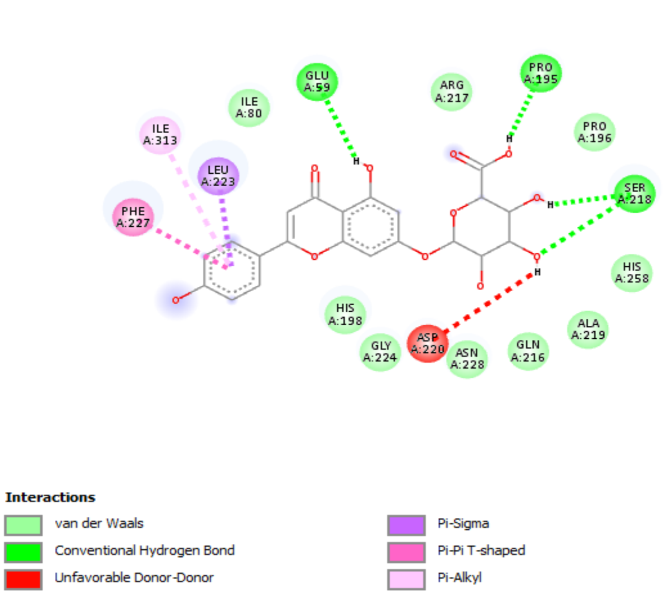

Apigenin 7-O-glucuronide

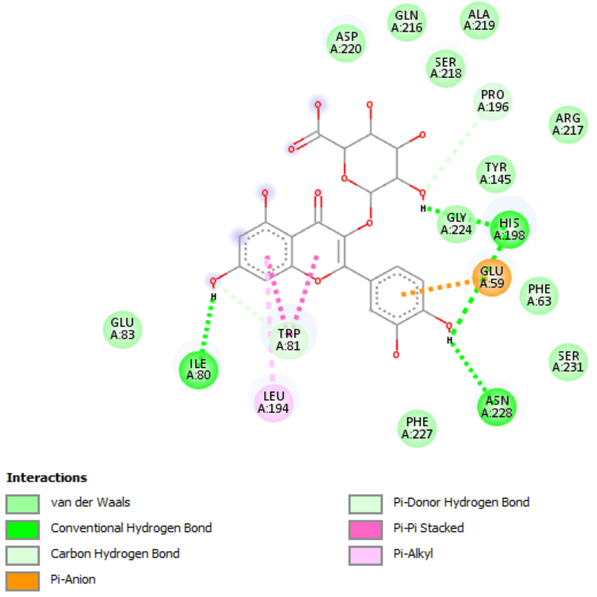

Quercetin-3-O-glucuronide

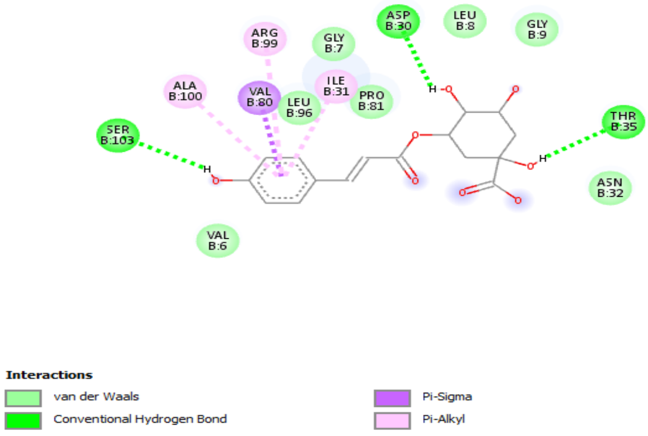

3-p-Coumaroylquinic acid

Figure S5. 2D view of the conformations of different interactions between the three inhibitors in the 6QXS active site.

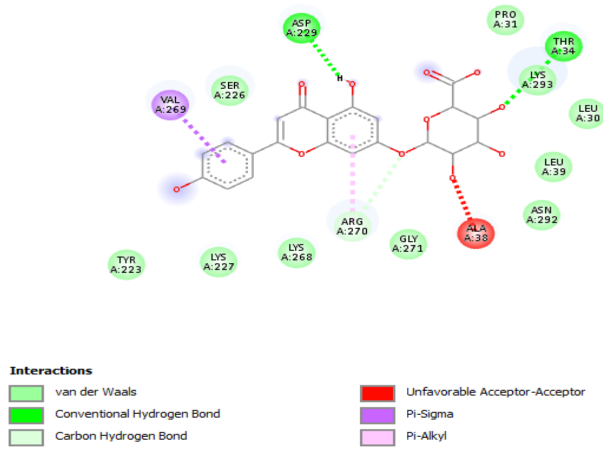

Apigenin 7-O-glucuronide

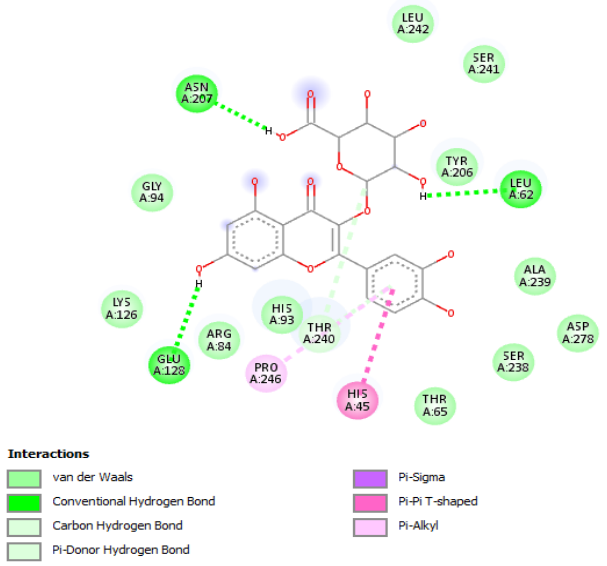

Quercetin-3-O-glucuronide

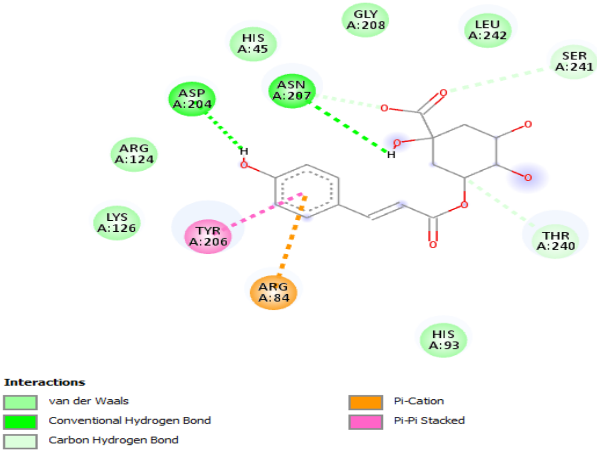

3-p-Coumaroylquinic acid

Figure S6. 2D view of the conformations of different interactions between the three inhibitors in the 1AOD active site.
